# Reduced Environmental Stimulation Therapy (REST) in methamphetamine use disorder: a pilot study

**DOI:** 10.1016/j.abrep.2025.100651

**Published:** 2025-12-13

**Authors:** Emily M. Choquette, McKenna Garland, Gregory Morrissey, Nicolas Wilzok, Raminta Wilson, Abhinita Premkumar, Jennifer L. Stewart, Sahib S. Khalsa

**Affiliations:** a Laureate Institute for Brain Research (LIBR), Tulsa, OK, USA; b Kendall College of Arts and Sciences, University of Tulsa, Tulsa, OK, USA; c Department of Psychotherapy and Psychosomatic Medicine, University Hospital Carl Gustav Carus Dresden, Technische Universität Dresden, Dresden, Germany; d Oxley College of Health & Natural Sciences, University of Tulsa, Tulsa, OK, USA; e Department of Psychiatry and Biobehavioral Sciences, Semel Institute for Neuroscience and Human Behavior, David Geffen School of Medicine, University of California at Los Angeles, Los Angeles, CA, USA

**Keywords:** Addiction, Interoception, Float Therapy, Reduced Environmental Stimulation Therapy

## Abstract

Existing treatments for methamphetamine use disorder (MUD) are suboptimal in addressing withdrawal and preventing relapse. Models of addiction have proposed that negative affect plays an influential role in drug craving and relapse. Although Reduced Environmental Stimulation Therapy (REST) has been associated with attenuated negative affect in other psychiatric conditions, it has not been examined as an intervention for MUD. To address this gap, the present study investigated the feasibility, tolerability, and safety of a single session of REST via floatation pool (pool-REST) in treatment-enrolled individuals with MUD. In a within-subject crossover design, participants (*n* = 78) consented to complete one session each of pool-REST and chair-REST, an active comparator controlling for the effects of simple relaxation and expectation. Feasibility was assessed via completion rate, tolerability via duration of REST utilization, and safety via adverse event incidence. Secondary outcomes were assessed via measurement of self-reported affect, interoceptive sensation, and degree of stimulant craving. 74 % of all recruited participants (*n* = 58) completed both REST sessions, which were well-tolerated. There were no serious adverse events and participants provided more frequent endorsements of positive versus negative experiences. Although both REST conditions showed significant decreases in ratings of stress/anxiety, negative affect, and stimulant craving, pool-REST was associated with greater increases in positive affect, cardiorespiratory interoceptive awareness, heart rate, and systolic blood pressure than chair-REST. These results demonstrate that REST sessions are feasible, well-tolerated, and safe in individuals with MUD. Randomized control trials are warranted to explore potential salutary effects of pool-REST on MUD remission and recovery.

## Highlights

• REST was feasible, safe, and tolerable for individuals with MUD.
• Interoception and positive affect increased during pool-REST but not chair-REST.
• REST reduced stress/anxiety, negative affect, and drug craving.
• Pool-REST may represent a novel adjunctive treatment for MUD.

## 1 Introduction

Methamphetamine use disorder (MUD) is a condition associated with serious mental and physical health consequences, including heightened drug craving, relapse, overdose-related death, cardiovascular dysfunction, and comorbid mood and anxiety disorders (Christian et al., 2007, Jones et al., 2020a, May et al., 2020, Paulus and Stewart, 2020). According to the National Survey on Drug Use and Health, MUD past-year prevalence increased in the United States from 2 million in 2019 to 2.6 million people in 2023 (Substance Abuse and Mental Health Services Administration., 2020, Substance Abuse and Mental Health Services Administration., 2024) with methamphetamine-related treatment admissions also increasing from 15 % in 2007 to 26 % in 2018 (Jones et al., 2020b) Of individuals reporting past-year use, 53 % met criteria for amphetamine use disorder, 27 % used > 200 days, and 22 % injected methamphetamine (Jones et al., 2020a). There are currently no FDA-approved pharmacological treatments for MUD cessation (Paulus & Stewart, 2020). Moreover, there is insufficient evidence that extant medications can effectively treat withdrawal symptoms and promote abstinence in individuals with MUD (Acheson et al., 2023). Though non-pharmaceutical interventions such as behavioral, psychological, and group therapy have demonstrated efficacy (AshaRani et al., 2020), the high rate of MUD relapse (61 % within 12 months; Brecht & Herbeck, 2014) reveals a need to explore additional interventions that directly target the processes associated with relapse.

Influential models of addiction highlight the potential role that negative reinforcement learning plays in relapse, such that stress and negative affect increase drug craving, thereby increasing the likelihood of future drug-use behaviors in spite of negative consequences (Koob & ., 2013, Koob and Volkow, 2016). According to these models, individuals may persist with methamphetamine use to avoid aversive states (e.g., withdrawal symptoms, anxiety, or depression). Additionally, disruptions in interoception, the process by which the nervous system senses and interprets internal body signals to guide motivated behavior (Khalsa et al., 2018), is thought to contribute to both drug craving and relapse (Paulus and Stewart, 2014, Volkow et al., 2019). For example, individuals with stimulant (amphetamine and cocaine) use disorders self-report lower trait interoceptive awareness than healthy individuals despite exhibiting amplified cardiac sensation intensity and lower insula activation during interoceptive attention, an effect inversely associated with drug use recency (Stewart et al., 2020). Given the absence of efficacious pharmacotherapies, these findings suggest a potential role for non-pharmacologic MUD interventions targeting interoception.

Floatation-REST (Reduced Environmental Stimulation Therapy) is a mind–body intervention that decreases exteroceptive input to the nervous system and has been shown to reduce anxiety/stress and increase cardiorespiratory interoception in individuals with mood, anxiety, and/or eating disorders (Choquette et al., 2023, Feinstein et al., 2018a, Feinstein et al., 2018b, Jonsson and Kjellgren, 2016, Khalsa et al., 2020). It has a well-established safety profile in these conditions (Feinstein et al., 2018b, Garland et al., 2023, Khalsa et al., 2020) but has not been studied in MUD. Given the therapeutic improvements of interoception observed during floatation-REST and the high comorbidity between anxiety and substance use disorders (Stewart & Conrod, 2008), it seems plausible that REST may lower aversive states and drug craving in individuals with MUD. Thus, the primary aim of the present pilot study was to investigate the feasibility, tolerability, and effects of floatation-REST in individuals with MUD.

Utilizing a within-subjects crossover design, treatment-seeking participants with MUD completed two counterbalanced REST sessions: one in a floatation pool (pool-REST), and one in an active comparator condition (chair-REST), aiming to control for the effects of expectancy and simple relaxation (as in Garland et al., 2023). Feasibility was assessed via session completion rates, tolerability via duration of REST utilization, and safety via incidence of serious or non-serious adverse events and ratings of positive and negative effects experienced during each session. We hypothesized that: (1) pool- and chair-REST completion rates would be high and unaffected by condition counterbalancing order; (2) pool- and chair-REST conditions would have similar session durations and be associated with few adverse events and low perceived negative side effects, and (3) pool-REST would demonstrate higher positive side effect ratings and effects on cardiorespiratory interoception than chair-REST, while resulting in greater reductions in subjective levels of stress/anxiety, drug craving, and negative affect.

## 2 Materials and Methods

### 2.1 Participant Recruitment

Treatment-enrolled adult participants were recruited from two Tulsa-area residential addiction recovery programs from March 2022 to November 2023. Inclusion criteria were: (1) current DSM-5 diagnosis of MUD; (2) > 2 weeks of treatment completion and abstinence at the time of enrollment; (3) English proficiency; (4) ability to complete informed consent and all study requirements. Exclusion criteria were: (1) history of DSM-5 schizophrenia spectrum, bipolar I, and other psychotic disorders; (2) consumption of antihistamines causing drowsiness or stimulating substances (e.g., caffeine) within two hours of REST sessions; (3) any unstable liver conditions or renal insufficiency, glaucoma, diabetes, significant and unstable cardiac, vascular, pulmonary, gastrointestinal, endocrine, neurologic, hematologic, rheumatologic, or metabolic disturbance; (4) a positive alcohol breathalyzer and/or a drug-positive urine test at either session; (5) unwillingness or inability to complete major aspects of the study protocol. Participants were required to be stably medicated for at least 48 h prior to each session. Psychiatric diagnoses were verified by a member of the Laureate Institute for Brain Research (LIBR) assessment team via a structured clinical interview employing the MINI (Sheehan et al., 1998). The current study was IRB approved by the WCB IRB (#20220646) and performed in accordance with Declaration of Helsinki. All volunteers provided written informed consent prior to study participation and received compensation via a ClinCard ($20 for completing baseline self-report measures and $40 for each REST session, for a total possible compensation of $100).

### 2.2 Study design

After obtaining informed consent, participants were randomly assigned to REST interventions in counterbalanced order: chair-REST or pool-REST (up to one hour each). Intervention sequences were placed in sealed envelopes by a staff member not otherwise involved in the study and the order was not revealed to either the participant or the experimenter until the day of the first session. REST sessions were completed on separate days scheduled 2 to 14 days apart (*M* = 5.68 days, SD = 3.15 days). Participants completed trait baseline measures prior to randomization (see Supplemental Information). Before each REST session, participants underwent urine drug screen and alcohol breathalyzer testing. They subsequently completed state-dependent measures of anxiety (Spielberger et al., 1983), drug craving (Sussner et al., 2006), positive/negative affect (Watson & Clark, 1994) and interoceptive awareness (Feinstein et al., 2018b), as well as vital sign measurement collected with a CASMED 750 device (CAS Medical Systems Inc) before and after each REST session. Participants sat for at least ten minutes before vital signs were collected. After each session, an assessment of potential adverse effects (Feinstein et al., 2018a, Feinstein et al., 2018b, Garland et al., 2023) was administered. Finally, participants underwent a debriefing interview in which they were given the opportunity to openly discuss their thoughts about the experience. Their spoken answers were recorded and later manually transcribed. A time of events table is available in the supplement (Supplemental Table S1). All surveys were obtained electronically using REDCap (Harris et al., 2019).

### 2.3 Experimental and active comparator conditions

**Fig. 1:**
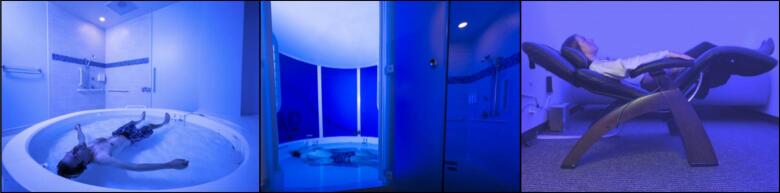
REST Environments. Pictured from left to right: pool-REST in an open pool, pool-REST in an enclosed pool, chair-REST. Each pool room was equipped with a private restroom, changing area, and shower. Participants were instructed to shower prior to entering the pool. In both conditions, ambient music played for the first eight minutes of the session. For the remainder of the hour, participants in both conditions had control over a blue LED light that they could turn on and off. With the lights off, the pool-REST environment was sound- and light-proof, with the water/air temperature set to 95 °F and 93.5 °F respectively, approximately matching that of the skin surface. The chair-REST environment was located in a quiet (though not sound or light proof) temperature-controlled room set to approximately 70 °F. An intercom system allowed for continuous communication between the participant and the experimenter throughout both REST Sessions. At the end of the REST session, the participant was cued that the session had ended with onset of music and/or lights. After the pool-REST session, participants took a second shower, and dried and redressed themselves. This figure is similar but not identical to original image used in Fig. 2 of Garland et al. (2023); the purpose of this image is to illustrate different REST environments. (For interpretation of the references to colour in this figure legend, the reader is referred to the web version of this article.)

Fig. 1 illustrates the pool-REST (experimental condition) and chair-REST (active comparator) session environments. Prior to their pool-REST session, participants chose between a “closed” domed pool and an “open” undomed pool (Floataway Inc). Conditions in both pools (i.e., temperature, humidity, specific gravity, and acidity) were kept within the same range. During the pool-REST session, participants floated effortlessly in a supine position in a pool of water highly concentrated with Epsom salt. Detailed pool specifications have been previously described (Feinstein et al., 2018a). Chair-REST was chosen to control for the effects of simple relaxation induced by lying down in a comfortable and quiet environment; here participants lay in the supine position in a Zero Gravity Chair (PC510, Classic Power, Series 2, Human Touch Inc.). The chair was composed of leather with cushioning made of memory foam. Participants could adjust the chair to recline to the position they found most comfortable.

### 2.4 Measures

Prior to randomization to ensure equivalent group allocation, participants completed trait baseline measures of anxiety (Spielberger et al., 1983, Taylor et al., 2007), depression (Kroenke et al., 2001), withdrawal symptoms (Srisurapanont et al., 1999), perceived stress (Cohen et al., 1983) and relaxation/arousal (Heide & Borkovec, 1983; see Supplemental Information).

2.4.1 Effects Checklist. A 43-item self-report measure was used to assess safety and subjective experiences occurring in each REST session (Feinstein et al., 2018a, Garland et al., 2023). Items assessed the presence of: (1) negative physical or psychological effects including itchiness, dry mouth, pain, anxiety/panic, flashbacks, suicidality, and hallucinations; and (2) positive physical or psychological effects (e.g., heightened feelings of joy, refreshment, serenity). Each item was rated on four-point scale (“none,” “mild,” “moderate,” or “extreme”) using the following instructions: “Did you notice or experience an increase in any of these items during or shortly after your float today? Please only mark items that showed an increase from your typical day-to-day experience.” For any item that had an endorsement other than “none,” participants were provided a free-response box to describe their experience in more detail. Specific negative symptom reports (strong emotional memories, flashbacks, heightened thoughts related to death, visual or auditory hallucinations, out-of-body experiences, feelings of detachment, loss of control over behavior, and flight of ideas/racing thoughts) receiving any endorsement other than “none” automatically resulted in a prompt with the following question: “Overall, was this a positive or negative experience?”. Items rated as “none” were coded as 0, negatively experienced items ranged from −3 (“extremely negative”) to −1 (“mildly negative”), and positively experienced items ranged from 1 (“mildly positive) to 3 (“extremely positive”).

2.4.2 State-Trait Anxiety Inventory (STAI-State). This 20-item questionnaire measures present-moment levels of anxiety (Spielberger et al., 1983). Items include typical anxiety symptoms and are rated on a four-point Likert scale ranging from “not at all” to “very much so.” Items were summed to create a total state-anxiety score, with higher scores representing higher levels of current anxiety.

2.4.3 Stimulant Craving Questionnaire (STCQ)–Brief. This 10-item scale adapted from a cocaine craving questionnaire (Sussner et al., 2006) assesses the degree of cravings and urges to use stimulant drugs (cocaine, amphetamine, methamphetamine) “right now” using a seven-point scale, with answers ranging from “strongly disagree” to strongly agree”. A composite score was generated by averaging all items, with higher scores indicating greater craving levels.

2.4.4 Positive and Negative Affect Schedule − Expanded Form (PANAS-X). This 60-item measure assesses affective states by asking participants to rate the extent to which they experience various emotions (Watson & Clark, 1994). Items are rated on a five-point Likert scale from “very slightly or not at all” to “extremely.” for the purposes of the current study, the “at the present moment” instructions were utilized.

2.4.5 Interoceptive awareness. To assess facets of interoceptive awareness, participants completed a self-report measure following each REST session (Feinstein et al., 2018b). The questions probed three visceral sensations (heartbeat, breath, and stomach/digestive system), and had participants provide retrospective ratings of the intensity, valence, and frequency of attentional focus on those sensations, using visual analog and Likert response scales. For intensity and attention questions, higher scores indicated more interoceptive intensity/attention. For valence questions, scores ranged from unpleasant to pleasant (0 to 100), with 50 indicating a neutral valence.

### 2.5 Statistical analysis

Feasibility was assessed via the percentage of participants randomized to each intervention arm that completed both sessions. Tolerability was determined by comparing REST condition session durations via a paired samples *t*-test. The number of adverse and serious adverse events and their relationship to the intervention was used to determine safety. Linear mixed effects (LME) modeling was used to predict adverse event magnitude ratings from experiences endorsed on the Effects Checklist, with REST condition (i.e., pool vs chair) as a within-subject fixed effect and subject as a random effect. Each model utilized an AR1 covariance structure. To interpret main effects and simple effects for interactions, post-hoc *t*-tests with Bonferroni-Holm corrections (Holm, 1979) were utilized.

LMEs also examined secondary clinical outcomes related to pre- versus post-session levels of state anxiety, drug craving, positive and negative affect, and physiological variables (systolic blood pressure, diastolic blood pressure, and heart rate). LME models included time and REST condition as within-subject fixed effects and subject as a random effect. Significant interactions were probed using *t*-tests with Bonferroni-Holm correction (Holm, 1979). To assess the influence of REST on interoceptive intensity, attention, and valence of visceral sensations (heartbeat, breath, and stomach/digestive system), nine paired samples *t*-tests were compared the two REST conditions. All analyses were performed in RStudio (Version 3.6.0; RStudio Team, 2020).

## 3 Results

### 3.1 Sample Characteristics/ baseline Questionnaire results

Sociodemographic and clinical characteristics for study completers (*n* = 58) are summarized in Table 1. Completers and non-completers did not differ on any demographic variables except for years of education (completers reported an average of 1.2 years more education than non-completers, see Supplemental Table S2). Medication information is listed in Supplemental Table S3 and S4**.** Counterbalancing order did not differ significantly on age, education, body mass index, baseline trait scores, sex or gender identity. Most participants identified as White (71 %), with smaller percentages identifying as American Indian or Alaska Native (5 %), Black or African American (2 %), or Multiple Races/‘Other’ Race (22 %). Four participants described their ethnicity as Hispanic (7 %).

**Table 1:** Participant Demographics, Diagnostic Comorbidities, and Entry Screening Scores.

		**Full** **Sample**	**Chair-** **Pool**	**Pool-** **Chair**	***F***	***df***	***p***	***α***
		(*n* = 58)	(*n* = 33)	(*n* = 25)				
**Demographics**								
Sex					*χ^2^* = 0.74	1	0.39	
	Male, *N* (%)	23 (40)	11 (33)	12 (48)				
	Female, *N* (%)	35 (60)	22 (67)	13 (52)				
Gender Identity					*χ^2^* = 2.44	2	0.29	
	Man, *N*(%)	23 (40)	11 (33)	12 (48)				
	Non-binary/Genderfluid, *N* (%)	3 (5)	1 (3)	2 (8)				
	Woman, *N* (%)	32 (55)	21 (64)	11 (44)				
Age		35.9 (8.0)	37.2 (8.8)	34.3 (6.5)	1.86	1	0.18	
Years of Education		12.4 (1.7)	12.5 (1.7)	12.2 (1.7)	0.32	1	0.57	
BMI		29.4 (5.0)	29.4 (5.4)	29.4 (4.5)	0.00	1	0.99	
Race/Ethnicity, *N* (%)					0.74	1	0.39	
	White	48 (83)	29 (88)	19 (76)				
	Black/African American	4 (7)	4 (12)	0 (0)				
	Am. Indian/Alaskan Native	9 (15)	5 (15)	4 (16)				
	Asian/Pacific Islander	1 (2)	0 (0)	1 (4)				
	Hispanic	4 (7)	1 (3)	3 (12)				
	Unspecified	6 (10)	2 (6)	4 (16)				
Diagnosis, *N* (%)								
	Amphetamine Use Disorder	58 (100)	33 (100)	25 (100)				
	Cannabis Use Disorder	18 (31)	9 (27)	9 (36)				
	Opioid Use Disorder	18 (31)	11 (33)	7 (28)				
	Alcohol Use Disorder	10 (17)	4 (12)	6 (24)				
	Cocaine Use Disorder	4 (7)	3 (9)	1 (4)				
	Sedative Use Disorder	4 (7)	2 (6)	2 (8)				
	Hallucinogen Use Disorder	1 (2)	1 (3)	0 (0)				
	Antisocial Personality Disorder	12 (21)	8 (24)	4 (16)				
	Major Depressive Disorder	14 (24)	9 (27)	5 (20)				
	Panic Disorder	3 (5)	3 (9)	0 (0)				
	Generalized Anxiety Disorder	1 (2)	1 (3)	0 (0)				
	Posttraumatic Stress Disorder	1 (2)	1 (3)	0 (0)				
**Self-Report Trait Measures**								
ASI-3		17.3 (14.2)	16.8 (14.4)	18 (14.2)	0.11	1	0.75	0.94
STAI-Trait		40.6 (13.1)	41.7 (14.1)	39.1 (11.6)	0.54	1	0.47	0.95
PHQ-9		5.6 (5.2)	5.62 (5.7)	5.60 (4.7)	0.00	1	0.99	0.87
AWQ		13.4 (7.6)	13.1 (7.8)	13.9 (7.4)	0.17	1	0.68	0.84
PSS		15.2 (7.2)	15.5 (7.1)	14.8 (7.4)	0.14	1	0.71	0.91
RRAQ		16.3 (11.3)	16.6 (11.4)	15.8 (11.4)	0.07	1	0.79	0.92

*Note.* Numbers reflect means and standard deviation unless otherwise indicated. BMI = Body Mass Index; ASI-3 = Anxiety Sensitivity Index-3; STAI-Trait = State Trait Anxiety Inventory- Trait Version; PHQ-9 = Patient Health Questionnaire-9; AWQ = Amphetamine Withdrawal Questionnaire; PSS = Perceived Stress Scale; RRAQ = Reactions to Relaxation and Arousal Questionnaire RRAQ. As individuals were able to self-identify with multiple races/ethnicities, percentage totals will be > 100 %.

### 3.2 Primary outcomes

**Fig. 2:**
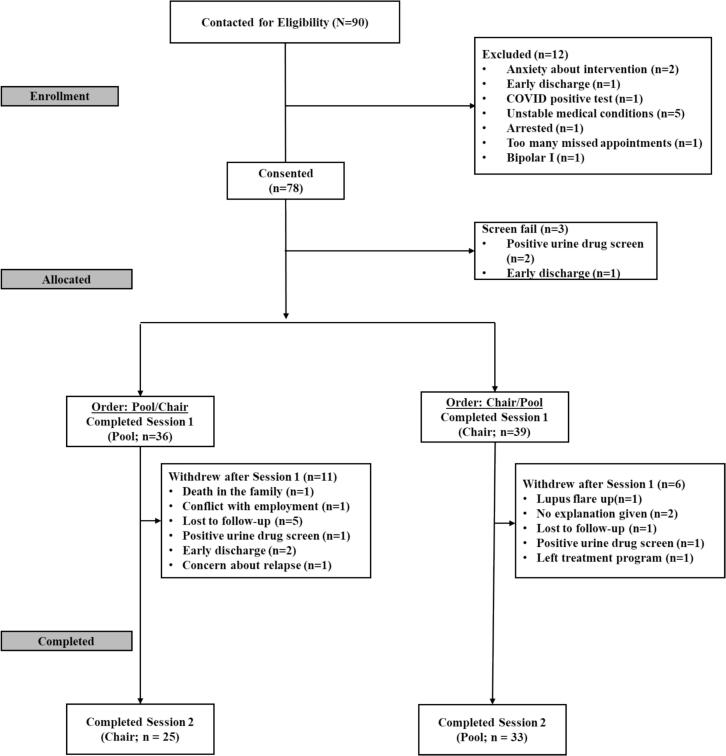
CONSORT Diagram.

**3.2.1 Feasibility.** 78 individuals consented to participate in the study, and 75 of those participants were randomized to a counterbalancing order (see Fig. 2 for CONSORT Diagram). 58 participants completed all phases of the study (74 %). Participant retention following discharge from inpatient treatment during participation was limited, with 12 out of 16 of early discharges withdrawing or being lost to follow-up. Of the participants assigned to complete the pool-REST condition first, 69 % completed both sessions (25/36 participants); whereas 85 % assigned to complete chair-REST first completed both sessions (33/39 participants; χ^2^(2) = 2.46, *p* = 0.12). Based on our interest in evaluating differential effects of pool-REST vs the chair-REST active comparator, subsequent analyses only included participants who completed both sessions (*n* = 58).

**3.2.2 Tolerability.** A paired samples *t*-test of within-session tolerability (i.e., session duration), revealed no significant differences across REST environments (*t*(57) = -1.60, *p* = 0.12), with mean session durations of 58.05 min (*SD* = 6.94) in the chair-REST environment and 56.67 min (*SD* = 7.60) in the pool-REST environment.

**3.2.3 Safety.** Of the 75 participants to undergo at least one session, there were no serious adverse events requiring medical treatment. One mild adverse event occurred in which a participant reported difficulty breathing during a pool session, leading to early termination of the session. The participant had elevated blood pressure upon terminating the session, which returned to normal, but they ultimately elected to withdraw from the study.

**Fig. 3:**
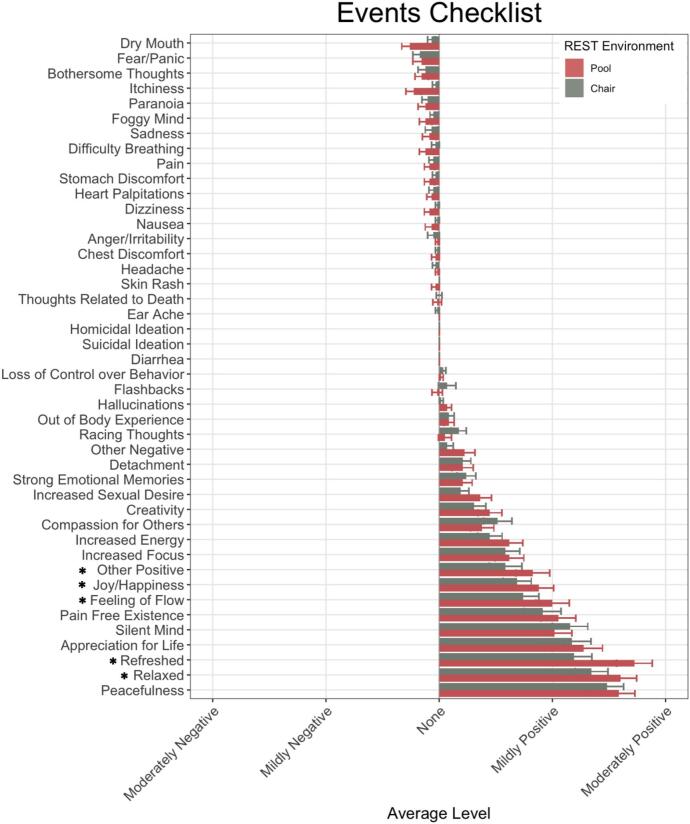
Primary Outcomes: Events Checklist Note. Event anchors ranged from “Extremely Negative” to “Extremely Positive” (anchors not shown). Asterisk (*) indicates significant difference in rating magnitude between REST environments.

Regarding the acutely experienced event profile, Fig. 3 shows the average magnitude rating for each event as a function of REST condition. A qualitative inspection of the data revealed that positive experiences were endorsed more commonly than negative ones and were also rated at higher levels of intensity, with average intensity ratings in the mild-to-moderate range for positive experiences and well-below mild for negative experiences. In the LME analysis of intensity ratings, there was a significant main effect of event (*F*(43, 4959) = 47.22, *p* < 0.0001, η_p_^2^ = 0.29), a marginally significant main effect of condition (*F*(1, 4959) = 3.67, *p* = 0.06, η_p_^2^ < 0.001), and a significant interaction between event and REST condition (*F*(43, 4959 = 1.88, *p* = 0.0004, η_p_^2^ = 0.02). Significant post-hoc comparisons (*p*s < 0.05) suggested that feelings of joy/happiness (*d* = 0.35), refreshment (*d* = 0.81), relaxation (*d* = 0.41), feelings of flow (*d* = 0.45), and “other positive experiences” (*d* = 0.39) were rated more positively in pool-REST than in chair-REST. There were no instances in which chair-REST was rated more positively than pool-REST. Qualitative inspection of free text entry responses for “other positive experiences” suggested common themes of gratitude and enlightenment.

### 3.3 Secondary outcomes

See Table 2 for Averaged Variables by time and condition.

**Table 2:** Means by condition and time for secondary outcomes.

	**Chair**		**Pool**		
	**Pre**	**Post**	**Pre**	**Post**	α*
	*M* (*SD*)	*M* (*SD*)	*M* (*SD*)	*M* (*SD*)	
STAI-S	35.3 (12.4)	29.3 (9.1)	35.6 (11.4)	29.5 (9.2)	0.93
Stimulant Craving	11.5 (6.0)	10.0 (4.3)	12.0 (6.9)	9.5 (5.4)	0.64
PANAS-X: Positive Affect	32.8 (10.2)	31.7 (10.6)	31.4 (11.2)	34.1 (9.7)	0.94
PANAS-X: Negative Affect	13.8 (4.7)	11.6 (3.6)	14.1 (5.4)	11.4 (3.0)	0.83
Systolic Blood Pressure	121.2 (12.3)	120.0 (13.3)	123.4 (13.2)	125.7 (13.8)	
Diastolic Blood Pressure	79.4 (8.3)	78.8 (10.9)	78.8 (10.6)	78.4 (10.6)	
Heart Rate	76.1 (12.7)	71.8 (11.1)	74.9 (13.6)	79.8 (12.6)	
**Intensity**					
Heartbeat	−	24.2 (27.2)	−	36.8 (32.2)	
Breath	−	32.9 (29.8)	−	54.7 (31.0)	
Stomach	−	24.7 (28.1)	−	31.3 (29.9)	
**Attention**					
Heartbeat	−	0.5 (0.9)	−	0.9 (1.1)	
Breath	−	0.7 (0.9)	−	1.6 (1.1)	
Stomach	−	0.5 (0.9)	−	0.6 (0.9)	
**Valence**					
Heartbeat	−	62.8 (20.8)	−	66.0 (19.5)	
Breath	−	68.2 (19.6)	−	66.7 (19.4)	
Stomach	−	57.6 (18.4)	−	61.2 (19.1)	

***Note.*** STAI-S*=* State-Trait Anxiety Inventory- State; PANAS-X = Positive and Negative Affective Schedule- Extended Version; *α calculated for full sample at time point one.

**Fig. 4:**
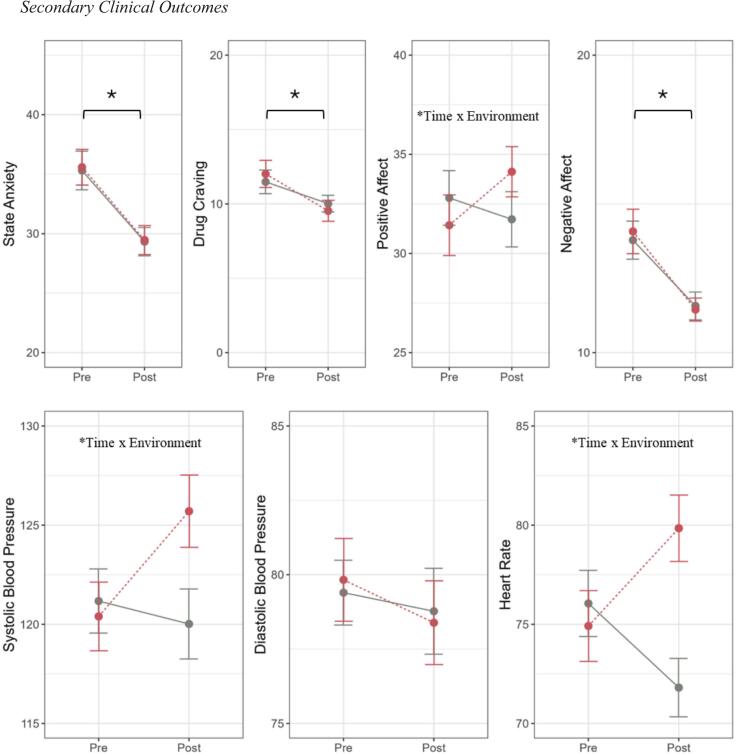
*Secondary Clinical Outcomes.* Line plots showing mean self-reported state anxiety, drug craving, positive affect, and negative affect scores and physiological variables (systolic, diastolic blood pressure and heart rate) before (‘pre’) and after (‘post’) REST sessions. Error bars reflect standard error of the mean. Asterisk (*) indicates a significant main effect of survey (*p<*.05), with lower ratings post-session.

**3.3.1 S**TAI**-**S**tate.** There was a significant main effect of time (*F*(1, 171) = 41.26, *p* < 0.0001, η_p_^2^ = 0.19), with pre- to post-session anxiety reductions across REST conditions (*p* < 0.0001, *d* = 0.89; Fig. 4a), and no differences between REST conditions

**3.3.2 Stimulant craving.** There was a significant main effect of time (*F*(1, 171) = 13.26, *p* = 0.0004, η_p_^2^ = 0.07), with pre- to post-session craving reductions across REST conditions (*p* = 0.0004, *d* = 0.58; Fig. 4b), and no differences between REST conditions

**3.3.3 PANAS-X: Positive affect.** A significant interaction between time and REST condition (*F*(1, 164) = 5.76, *p* = 0.02, η_p_^2^ = 0.03) indicated that positive affect showed a pre- to post-session increase in pool-REST (*p* = 0.02, *d* = 0.43) but not chair-REST (*p* = 0.26, *d* = 0.21; Fig. 4c)

**3.3.4 PANAS-X: Negative affect.** A significant main effect of time (*F*(1, 163) = 32.89, *p* < 0.0001, η_p_^2^ = 0.17) indicated that negative affect showed a significant pre- to post-session reduction across REST conditions (*p* < 0.0001, *d* = 0.79; Fig. 4d), and no differences between REST conditions.

**3.3.5 Physiological outcomes.** The LME analysis of systolic blood pressure (Fig. 4e) revealed significant main effects of time (*F*(1, 169) = 4.16, *p* = 0.04, η_p_^2^ = 0.02) and condition (*F*(1, 169) = 5.17, *p* = 0.02, η_p_^2^ = 0.03), which were qualified by a significant time-by-condition interaction (*F*(1, 169) = 9.59, *p* = 0.002, η_p_^2^ = 0.05). Simple effects testing showed systolic blood pressure increases pre- to post-session for pool-REST (*p* = 0.0004, *d* = 0.30) but not chair-REST (*p* = 0.37, *d* = 0.54). The LME analysis of diastolic blood pressure (Fig. 4f) revealed no significant main effects or interactions (all *p*s > 0.05).

The LME analysis of heart rate (Fig. 4g) revealed a significant main effect of condition (*F*(1, 169) = 8.50, *p* = 0.004, η_p_^2^ = 0.05) that was qualified by a time-by-condition interaction (*F*(1, 169) = 27.06, *p* < 0.0001, η_p_^2^ = 0.14). Simple effects tests suggested that heart rate increased from pre- to post-session in pool-REST (*p* < 0.0001, *d* = 0.79) but conversely decreased with time in chair-REST (*p* = 0.0006, *d* = 0.60).

**Fig. 5:**
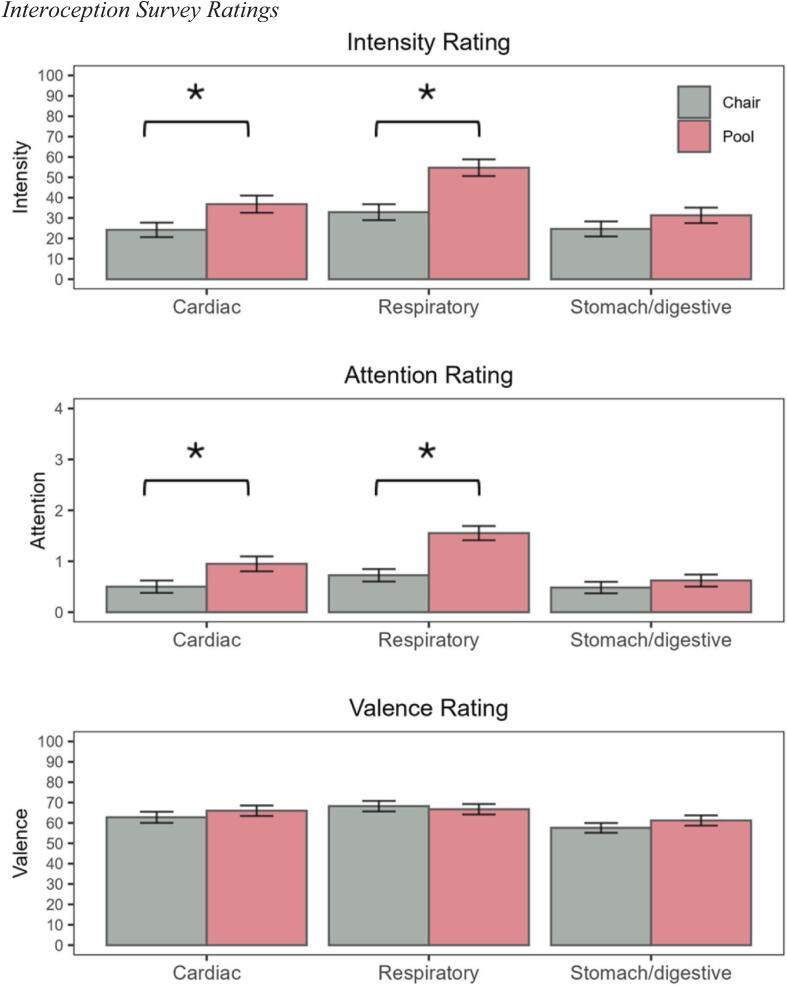
Interoception Survey Ratings. Mean retrospective self-ratings related to intensity (row 1), attention (row 2), and valence (row 3) of cardiovascular, respiratory, and gastrointestinal sensations. Error bars reflect standard error of the mean. Asterisk (*) indicates significant difference between REST environments (p < 0.05). Note: a valence score of 50 indicates a neutral rating.

**3.3.6 Interoceptive awareness.** Pool-REST evoked higher intensity ratings than chair-REST for heartbeat (*t*(57) = 2.94, *p* = 0.005) and breathing (*t*(56) = 4.72, *p* < 0.0001) sensations, but not for stomach/digestive sensations (*t*(57) = 1.45, *p* = 0.15; Fig. 5). A similar pattern emerged for attention ratings, whereby pool-REST elicited greater frequencies of attention towards the heartbeat (*t*(57) = 2.99, *p* = 0.004) and breath (*t*(57) = 5.52, *p* < 0.0001) than chair-REST. There were no condition differences for attention towards gastrointestinal sensations (*t*(57) = 0.97, *p* = 0.34) or valence ratings of heartbeat (*t*(56) = 1.09, *p* = 0.28), breath (*t*(56) = 0.48, *p* = 0.64), or stomach/digestive sensations (*t*(56) = 1.24, *p* = 0.22).

## 4 Discussion

The present study indicates that single sessions of pool-REST and chair-REST are tolerable, feasible, and safe for participants with MUD. With respect to feasibility, 74 % of participants completed both sessions, a number consistent with average dropout rates from substance use disorder treatment programs (Lappan et al., 2020). In addition, both REST conditions were tolerable, with most participants utilizing the entire allotted time of 60 min. Finally, our results suggest that REST is safe, as evidenced by: (1) only one mild adverse event reported; and (2) positive events experienced more frequently and at higher levels of intensity than negative events.

The low retention of study participants post-discharge from inpatient treatment suggests that REST interventions may be most feasible for individuals actively enrolled in treatment. Participants completing the pool-REST condition first were somewhat more likely to withdraw than those completing chair-REST, although this difference was not statistically significant. As many of these participants were lost to follow-up, it was not possible to determine the precise reasons for withdrawal; however, these participants rated their pool-REST experience as pleasant. Taken in combination with the relatively low prevalence of adverse events, our findings suggest that unpleasant pool-REST experiences likely did not contribute to differential withdrawal rates.

With respect to secondary outcomes, analysis of pre- to post-REST session questionnaires showed increases in positive affect immediately following pool-REST, but not chair-REST. Specifically, joy/happiness, refreshment, relaxation, flow, gratitude, and enlightenment were experienced more intensely in pool-REST than in chair-REST. While there was an overall REST effect on reduced stimulant craving and anxiety, this did not differ by condition. Given the aforementioned links among negative affect, drug craving, and relapse (Koob & ., 2013, Koob and Volkow, 2016), our results suggest that pool-REST may support recovery of MUD patients by increasing positive affect and reducing negative affect. Future research should explore whether the observed effects with pool-REST may be combined with other evidence-based forms of non-pharmacologic therapies to enhance treatment outcomes (e.g., float-assisted psychotherapy).

Consistent with previous studies in clinical populations (Feinstein et al., 2018a, Khalsa et al., 2020), pool-REST amplified the intensity of and attention to cardiorespiratory sensations. These increases in subjective intensity were accompanied by increased heart rate and systolic blood pressure but were not interpreted as negative by participants. In contrast to previous studies showing reductions in blood pressure (Feinstein et al., 2018b, Flux et al., 2022, Jacobs et al., 1984, Turner et al., 1989) during and after pool-REST, our findings are consistent with those of Khalsa and colleagues (Fig. 6 in Khalsa et al., 2020) who found a U-shaped relationship between systolic and diastolic blood pressure over time such that blood pressure decreased early during the float, remained stable, and then increased after the float. Prior heart rate findings as a function of pool-REST have also been mixed, with: (1) reductions evident during but not after pool-REST (Forgays & Belinson, 1986); (2) reductions during pool-REST compared to an active comparator (O’Leary & Heilbronner, 1990); and (3) no condition differences in heart rate (Flux et al., 2022). The variability in these results may be explained by the timing of physiological recording as well as the different clinical groups being studied (e.g., MUD versus eating or anxiety disorders). The protocol for this study was most consistent with Khalsa and colleagues (2020) in which post-session measurements were recorded after the participant had exited pool-REST and showered, which may have contributed to blood pressure and heart rate increases. Alternatively, it seems plausible that blood pressure/heart rate and subjective cardiorespiratory interoceptive increases evident in the pool-REST condition could have contributed to the positive affect changes evident in this context. Additional research is warranted to more comprehensively examine the relationships between changes in interoceptive and affective changes occurring in pool-REST. On the whole, given the well-known sympathomimetic cardiovascular effects of stimulants and the documented interoceptive deficits in stimulant users reflecting altered processing of cardiac sensations (Koob & ., 2013), the pool-REST environment could present a novel state intervention for reducing craving while enhancing interoceptive awareness and positive affect, thereby attenuating negative reinforcement mechanisms thought to drive future relapse.

### 4.1 Limitations

This study has several limitations. First, our findings were in treatment-enrolled individuals who were in early methamphetamine abstinence. Thus, it is unclear whether the observed safety, tolerability, and beneficial effects of REST would apply to individuals in the active stages of methamphetamine use or methamphetamine withdrawal. Second, we focused on short-term safety, feasibility, and tolerability. Longitudinal data were not collected to assess future relapse or the potential impact of multiple pool-REST sessions. Identification of the duration of REST effects, the ideal session number, and influence on long-term prognosis remain open questions. It is also unknown whether the efficacy of pool-REST might be favorably modulated (or otherwise) when combined with interventions such as pharmaceutical agents or psychotherapy. Third, from an experimental point of view, the salubrious impacts of chair-REST on negative affect and craving suggest it may not be distinct enough an intervention from pool-REST to be an ideal active comparator, which may explain the encouraging results seen in both interventions. Finally, there is still a limited understanding of the mechanistic underpinnings of REST.

### 4.2 Conclusion

Further randomized controlled trials are warranted to investigate the potential effects of pool-REST on remission and recovery from methamphetamine use disorders.

Role of Funding Sources.

This study was supported by the Laureate Institute for Brain Research.

## CRediT authorship contribution statement

**Emily M. Choquette:** Writing – review & editing, Writing – original draft. **McKenna Garland:** Writing – review & editing, Formal analysis. **Gregory Morrissey:** Writing – review & editing, Writing – original draft, Investigation, Formal analysis, Data curation, Conceptualization. **Nicolas Wilzok:** Data curation. **Raminta Wilson:** Formal analysis, Data curation. **Abhinita Premkumar:** Investigation, Formal analysis. **Jennifer L. Stewart:** Writing – review & editing, Supervision, Methodology, Funding acquisition, Formal analysis, Conceptualization. **Sahib S. Khalsa:** Writing – review & editing, Supervision, Methodology, Funding acquisition, Formal analysis, Conceptualization.

## Declaration of competing interest

The authors declare that they have no known competing financial interests or personal relationships that could have appeared to influence the work reported in this paper.

## Data Availability

Data will be made available on request.
