# Supplementary material for: Reduced Environmental Stimulation Therapy (REST) in methamphetamine use disorder: a pilot study
Source: Addict Behav Rep. 2025 Dec 13;23:100651. doi: 10.1016/j.abrep.2025.100651 (PMC12813051; doi:10.1016/j.abrep.2025.100651)
Supplement: Supplementary Data 1 [file mmc1.docx]

**SUPPLEMENTAL MATERIALS**

**Reduced Environmental Stimulation Therapy (REST) in Methamphetamine Use Disorder: A Pilot Study**

Emily M. Choquette, PhD^1^; McKenna Garland, PhD^1,2^; Gregory Morrissey, MD^1^; Nicolas Wilzok^3^; Raminta Wilson, MD, MPH^1^; Abhinita Premkumar, BS^1^; Jennifer L. Stewart, PhD^1,4^;

Sahib S. Khalsa, MD, PhD^1,5^*

^1^Laureate Institute for Brain Research (LIBR), Tulsa, Oklahoma, USA

^2^Kendall College of Arts and Sciences, University of Tulsa, Tulsa, Oklahoma

^3^Department of Psychotherapy and Psychosomatic Medicine, University Hospital Carl Gustav Carus Dresden, Technische Universität Dresden, Dresden, Germany

^4^Oxley College of Health & Natural Sciences, University of Tulsa, Tulsa, Oklahoma, USA

^5^Department of Psychiatry and Biobehavioral Sciences, Semel Institute for Neuroscience and Human Behavior, David Geffen School of Medicine, University of California at Los Angeles, Los Angeles, CA

*Corresponding author

**Table S1**

Time of Events

| Procedures | Screening | Visit 1 (Float 1) | Visit 2 (Float 2) |
| --- | --- | --- | --- |
| LIBR Screening Informed consent* | X |  |  |
| Demographics* | X |  |  |
| Medical History* | X |  |  |
| Current Medications* | X |  |  |
| Clinical Interview* | X |  |  |
| Providing the Pre-Float Checklist | X |  |  |
| Anxiety Sensitivity Index (ASI-3) |  | X |  |
| Amphetamine Withdrawal Questionnaire (AWQ) |  | X |  |
| Patient Health Questionnaire (PHQ-9) |  | X |  |
| Perceived Stress Scale (PSS) |  | X |  |
| Reactions to Relaxation and Arousal Questionnaire (RRAQ) |  | X |  |
| State-Trait Anxiety Inventory (STAI-Trait form) |  | X |  |
| Study-specific informed consent |  | X |  |
| Vital signs – before and after each float |  | X | X |
| Urine drug screen |  | X | X |
| Breathalyzer test |  | X | X |
| Order Assignment |  | X |  |
| Administer assigned intervention |  | X | X |
| Debriefing Interview |  | X | X |
| Effects Checklist |  | X | X |
| Positive and Negative Affect Schedule - Expanded Form (PANAS-X; *Pre-Post Session*) |  | X | X |
| State-Trait Anxiety Inventory (STAI-State form; *Pre-Post Session*) |  | X | X |
| Stimulant Craving Questionnaire (STCQ)–Brief *(Pre-Post Session)* |  | X | X |
| Adverse Event Monitoring |  | X | X |

*Note.* *Covered in separate LIBR screening protocol approved by WCG (# 20101611).

**Table S2**

*Demographic Variables for Completers and Non-completers*

|  | **Completer** | **Non-Completer** | ***F*** | ***df*** | ***p*** |
| --- | --- | --- | --- | --- | --- |
|  | (*n*=58) | (*n*=20) |  |  |  |
| **Condition** | *N* (%) | *N* (%) | *χ^2^*= 1.67 | 1 | 0.20 |
| Chair | 33 (84.6%) | 6 (15.4%) |  |  |  |
| Pool | 25 (69.4%) | 11 (30.6%) |  |  |  |
| **Demographics** |  |  |  |  |  |
| Sex |  |  | *χ^2^* = 0.81 | 1 | 0.37 |
| Male, *N* (%) | 23 (40) | 9 (45) |  |  |  |
| Female, *N* (%) | 35 (60) | 7 (35) |  |  |  |
| Unavailable *N* (%) | 0 (0) | 4 (20) |  |  |  |
| Gender Identity |  |  | *χ^2^* = 3.07 | 2 | 0.21 |
| Man, *N* (%) | 23 (40) | 12 (60) |  |  |  |
| Non-binary/Genderfluid*, N* (%) | 3 (5) | 0 (0) |  |  |  |
| Woman, *N* (%) | 32 (55) | 8 (40) |  |  |  |
| Age | 35.9 (8.0) | 35.1 (11.1) | 0.14 | 1 | 0.71 |
| Years of Education | 12.4 (1.7) | 11.2 (2.2) | 5.4 | 1 | 0.03* |
| BMI | 29.4 (5.0) | 28.5 (5.0) | 0.50 | 1 | 0.49 |
| Race/Ethnicity, *N* (%) |  |  | 0.33 | 1 | 0.57 |
| White | 48 (83) | 17 (85) |  |  |  |
| Black/African American | 4 (7) | 2 (10) |  |  |  |
| Am. Indian/Alaskan Native | 9 (15) | 4 (20) |  |  |  |
| Asian/Pacific Islander | 1 (2) | 0 (0) |  |  |  |
| Hispanic | 4 (7) | 1 (0.5) |  |  |  |
| Unspecified | 6 (10) | 0 |  |  |  |
| Diagnosis, *N* (%) |  |  |  |  |  |
| Amphetamine Use Disorder | 58 (100) | 20 (100) |  |  |  |
| Cannabis Use Disorder | 18 (31) | 8 (40) |  |  |  |
| Opioid Use Disorder | 18 (31) | 6 (30) |  |  |  |
| Alcohol Use Disorder | 10 (17) | 8 (40) |  |  |  |
| Cocaine Use Disorder | 4 (7) | 3 (15) |  |  |  |
| Sedative Use Disorder | 4 (7) | 4 (20) |  |  |  |
| Hallucinogen Use Disorder | 1 (2) | 0 (0) |  |  |  |
| Antisocial Personality Disorder | 12 (21) | 6 (30) |  |  |  |
| Major Depressive Disorder | 14 (24) | 3 (15) |  |  |  |
| Panic Disorder | 3 (5) | 2 (10) |  |  |  |
| Generalized Anxiety Disorder | 1 (2) | 1 (5) |  |  |  |
| Posttraumatic Stress Disorder | 1 (2) | 1 (5) |  |  |  |

| **Table S3**  *Participant Medications* | |  |
| --- | --- | --- |
|  | | *N* (%) |
| Taking at least one psychiatric medication | | 40 (69.0) |
|  | Opioid agonist/antagonist ^1^ | 12 (20.7) |
|  | Melatonin agonist ^2^ | 11 (19.0) |
|  | Histamine antagonist ^3^ | 14 (24.1) |
|  | GABA modulator ^4^ | 2 (3.4) |
|  | Serotonin modulator ^5^ | 27 (46.6) |
|  | Glutamate modulator ^6^ | 9 (15.5) |
|  | Dopamine modulator ^7^ | 9 (15.5) |
|  | Norepinephrine modulator ^8^ | 19 (32.8) |
|  | Muscarinic antagonist^9^ | 1 (1.7) |
|  | Unspecified anxiolytic | 1 (1.7) |
|  | Unspecified psychotropic | 1 (1.7) |
| Not taking any psychiatric medication | | 18 (31.0) |
|  | | *M* (*SD*) |
| Psychiatric medications per participant | | 2.0 (1.7) |

^1^buprenorphine/naloxone, naloxone

^2^melatonin supplementation

^3^hydroxyzine, mirtazapine

^4^baclofen^ag^, topiramate

^5^buspirone^pag^, citalopram^ri^, duloxetine^ri^, escitalopram^ri^, fluoxetine^ri^, mirtazapine^ant^, olanzapine^ant^, quetiapine^ant^, risperidone^ant^, sertraline^ri^, trazodone^ant^, venlafaxine^ri^

^6^gabapentin^cb^, oxcarbazepine^cb^, topiramate^ant^

^7^bupropion^ri/re^, olanzapine^ant^, quetiapine^ant^, risperidone^ant^

^8^bupropion^ri/re^, clonidine^ag^, duloxetine^ri^, mirtazapine^ant^, prazosin^ant^, quetiapine^ant^, risperidone^ant^, trazodone^ant^, venlafaxine^ri^

^9^benzatropine

Abbreviations: ^ag^ agonist, ^ant^ antagonist, ^pag^ partial agonist, ^cb^ channel blocker, ^ri^ reuptake inhibitor, ^re^ releaser

| **Table S4**  *Participant Non-Psychiatric Medications* | |  |
| --- | --- | --- |
|  | | *N* (%) |
| Taking at least one non-psychiatric medication | | 27 (46.6) |
|  | Acid reducer^1^ | 1 (1.7) |
|  | Analgesic/Antipyretic^2^ | 1 (1.7) |
|  | Anticoagulant^3^ | 1 (1.7) |
|  | Antihistamine^4^ | 1 (1.7) |
|  | Antihypertensive^5^ | 10 (17.2) |
|  | Antimicrobial^6^ | 6 (10.3) |
|  | Corticosteroid^7^ | 3 (5.2) |
|  | Diuretic^8^ | 1 (1.7) |
|  | Expectorant^9^ | 1 (1.7) |
|  | Hormone Modulator^10^ | 2 (3.4) |
|  | Laxative^11^ | 1 (1.7) |
|  | Non-Steroidal Anti-Inflammatory Drug^12^ | 6 (10.3) |
|  | Supplement^13^ | 10 (17.2) |
|  | Vasoconstrictor^14^ | 1 (1.7) |
| Not taking any non-psychiatric medication | | 31 (53.4) |
|  |  | *M* (*SD*) |
| Non-psychiatric medications per participant | | 0.9 (1.2) |

^1^omeprazole

^2^acetaminophen

^3^apixaban

^4^cetirizine

^5^amlodipine, atenolol, lisinopril, metoprolol, propranolol

^6^azithromycin, bictegravir/emtricitabine/tenofovir alafenamide, cephalexin, clindamycin, trimethoprim/sulfamethoxazole

^7^fluticasone, prednisone

^8^hydrochlorothiazide

^9^guaifenesin

^10^etonogestrel, finasteride, levothyroxine

^11^docusate

^12^celecoxib, diclofenac, ibuprofen, naproxen

^13^iron supplement, multivitamin, prenatal vitamin

^14^tamsulosin

**Supplemental Information**

**Baseline Self-Report Measures.** Measures of anxiety (Spielberger et al., 1983; Taylor et al., 2007), depression (Kroenke et al., 2001), withdraw symptoms (Srisurapanont et al., 1999), perceived stress (Cohen et al., 1983) and relaxation/arousal (Heide & Borkovec, 1983) were administered prior to the first REST session.

**The Anxiety Sensitivity Index** (**ASI-3;** Taylor et al., 2007) is an 18 item measure designed to assess the fear of arousal-related sensations, specifically along the dimensions/subscales of Physical, Cognitive, and Social Concerns. Each item is answered on a scale of 0-4 (“very little” to “very much”). Total score ranges from 0-72 with high scores indicating greater symptomatology. Cronbach’s α = 0.95 for full sample at baseline (*M* = 21.36, *SD* = 18.37).

The **State-Trait Anxiety Inventory** (**STAI-Trait form; S**pielberger et al., 1983) is a widely used psychometric instrument designed to assess an individual’s anxiety proneness. This measure has both a “state” subscale meant to measure temporary anxiety symptoms and a “trait” subscale meant to measure more long-standing anxiety proneness. Each subscale consists of 20 items using 4-point scales (“not at all” to “almost always”). The scale ranges from 20-80 with higher scores indicating more trait anxiety. Cronbach’s α = 0.95 for full sample at baseline (*M* = 40.57, *SD* = 13.06).

The **Patient Health Questionnaire** (**PHQ;** Kroenke et al., 2001) is a self-administered diagnostic instrument for common mental disorders. The PHQ-9 is the depression module, which scores each of the 9 DSM-IV criteria as “0” (not at all) to “3” (nearly every day). Scores of 1-4 are considered minimal depression, 5-9 mild depression, 10-14 moderate depression, 15-19 moderately severe depression and 20-27 severe depression. Cronbach’s α = 0.87 for full sample at baseline (*M* = 5.60, *SD* = 5.17).

**Amphetamine Withdrawal Questionnaire** (**AWQ;** Srisurapanont et al., 1999) is a 10 item scale in which participants indicate the extent to which they have experienced amphetamine withdrawal symptoms over the past 24 hours using a 5-point Likert scale (0 = not at all, 1 = a little, 2 = moderately, 3 = quite a bit, 4 = extremely). The scale ranges from 0-40 with greater scores indicating greater withdrawal symptoms. Cronbach’s α = 0.87 for full sample at baseline (*M* = 13.45, *SD* = 7.60).

**Perceived Stress Scale** (**PSS;** Cohen et al., 1983) is a psychological instrument used for measuring an individual’s perception of stress. It has become one of the most widely used instruments for measuring the degree to which situations in one’s life are appraised as stressful. The scale ranges from 0-40 with higher scores indicating higher levels of perceived stress. Cronbach’s α = 0.91 for full sample at baseline (*M* = 15.20, *SD* = 7.20).

The **Reactions to Relaxation and Arousal Questionnaire** (**RRAQ;** Heide & Borkovec, 1983) assesses one’s propensity to become more anxious during periods of relaxation, a paradoxical effect that has been found in some individuals with high levels of anxiety. Total scale scores range from 9 to 45. Cronbach’s α = 0.92 for full sample at baseline (*M* = 16.26, *SD* = 11.34).
